# Food Insecurity, Food Assistance, and Psychological Distress among University Students: Cross-Sectional Survey Western Australia, 2020

**DOI:** 10.3390/nu15112431

**Published:** 2023-05-23

**Authors:** Liyuwork Mitiku Dana, Janine Wright, Rebecca Ward, Jaya A. R. Dantas, Satvinder S. Dhaliwal, Blake Lawrence, Moira O’Connor, Sue Booth, Deborah A. Kerr, Christina M. Pollard

**Affiliations:** 1 School of Population Health, Faculty of Health Science, Curtin University, Bentley, WA 6102, Australia; liyuwork.dana@curtin.edu.au (L.M.D.); janine@ishar.org.au (J.W.); rebecca.e.ward@postgrad.curtin.edu.au (R.W.); jaya.dantas@curtin.edu.au (J.A.R.D.); s.dhaliwal@curtin.edu.au (S.S.D.); blake.lawrence@curtin.edu.au (B.L.); m.oconnor@curtin.edu.au (M.O.); d.kerr@curtin.edu.au (D.A.K.); 2 Curtin Health Innovation Research Institute, Faculty of Health Sciences, Curtin University, Perth, WA 6102, Australia; 3 Duke-NUS Medical School, National University of Singapore, College Road, Singapore 169857, Singapore; 4 Institute for Research in Molecular Medicine (INFORMM), Universiti Sains Malaysia, Jalan Inovasi, George Town 11800 USM, Malaysia; 5 Office of the Provost, Singapore University of Social Sciences, 463 Clementi Rd, Singapore 599494, Singapore; 6 Enable Institute, Curtin University, Bentley, WA 6102, Australia; 7 College of Medicine and Public Health, Flinders University, Sturt Rd, Bedford Park, Adelaide, SA 5042, Australia; sue.booth@flinders.edu.au

**Keywords:** food insecurity, food assistance, COVID-19 pandemic, university students, psychological distress, children

## Abstract

University students have been identified as a population sub-group vulnerable to food insecurity. This vulnerability increased in 2020 due to the COVID-19 pandemic. This study aimed to assess factors associated with food insecurity among university students and the differences between students with and without children. A cross-sectional survey of (n = 213) students attending one university in Western Australia measured food insecurity, psychological distress, and socio-demographic characteristics. Logistic regression analyses were conducted to identify factors associated with food insecurity. Forty-eight percent of students who responded to the survey had experienced food insecurity in 2020. International students who were studying in Australia were nine times more likely to experience food insecurity than domestic students (AOR = 9.13; 95% CI = 2.32–35.97). International students with children were more likely to experience food insecurity than international students without children (*p* < 0.001) and domestic students with (*p* < 0.001) or without children (*p* < 0.001). For each unit increase in depression level, the likelihood of experiencing food insecurity increased (AOR = 1.62; 95% CI = 1.12–2.33). Findings show a higher prevalence of food insecurity among international university students and students with children during the COVID-19 pandemic and that food insecurity was associated with higher levels of psychological distress. These findings highlight the need for targeted interventions to mitigate the risk of food insecurity among Australian university students, particularly among international students, students with children, and those experiencing psychological distress.

## 1. Introduction

Food insecurity expresses itself when there is a disruption to regular food consumption patterns due to financial or other resource limitations and represents a violation of the fundamental human right to access safe, adequate, and nutritious food [1]. The prevalence of food insecurity is increasing in many wealthy countries, largely due to the unequal distribution of resources [2,3]. Australia is reportedly one of the most food-secure countries in the world, with the government reporting a population prevalence of around five percent [3,4,5]. Some sub-population groups are at a higher risk of food insecurity; younger adults, low-income earners or un- or underemployed people, immigrants, and those living in rental accommodation [6,7,8,9]. The prevalence of food insecurity among tertiary education (university) students in economically rich countries is increasing, estimated to be between 13 and 48% of students [10,11,12].

The COVID-19 pandemic and mitigation strategies (e.g., stay-at-home orders and business closures) further exacerbated food insecurity [6,13,14]. Interrupted food supply chains and disruptions in economic resources, particularly among vulnerable population sub-groups (e.g., university students and low-income families with children), were reported in wealthy countries [14,15]. In Australia, states and territories took different approaches to contain the virus following the Federal Government’s nationwide lockdown in March 2020 [16]. The rapid social and economic changes had wide-ranging ramifications, including increasing the rate of unemployment during the early stages of the pandemic [17]. Most accommodation and food services industries were impacted, both major employers of young people and temporary visa holders [18].

The prevalence of food insecurity among university students increased during the pandemic [15,19,20,21,22,23]; however, most studies in the early stages of the pandemic were conducted in North America [19,20,21,22,23], and little was known about the factors correlated with food insecurity among university students in Australia. Those that were conducted did not explore the impact on students who were parents, and they used different instruments (e.g., with 1, 2, 6, or 8 items) that did not assess all aspects of the experiences of food insecurity [24,25]. The 18 item United States Department of Agriculture Food Security Survey Module (USDA-FSSM) is a validated and reliable measure that enables a comprehensive examination of household food insecurity experiences and their impact on children [26,27]. Addressing the research gap, the current study used the 18 item USDA-FSSM.

Food insecurity among university students is associated with a range of costly and preventable health consequences and poor academic performance [11,22,28,29,30,31]. Compared to students who are food secure, those experiencing food insecurity are more likely to report: limited physical activity [28]; consuming energy-dense nutrient-poor food [11] and fewer servings of fruits and vegetables [28,29]; disordered eating behaviors [30]; not enough sleep [28]; difficulty concentrating [10]; poor social connection [28,29]; emotional and behavioral problems [22,32]; and poor academic achievements [31,33].

There appear to be associations between food insecurity and mental health issues such as depression and suicide ideation in young adults [34,35]. Anxiety and stress have also been found to be associated with food insecurity; however, this relationship is not consistently reported. A positive relationship between food insecurity and anxiety and stress among university students has been reported [22,34], whereas some studies show no association [36]. There is also a dearth of information about the relationship between psychological distress and the severity of food insecurity among university students.

Given the increasing impact of disaster events on the extent and severity of food insecurity, particularly among vulnerable population sub-groups such as university students and those with children [37,38], and the potential consequences on health, social, and psychological distress, exploring the extent, severity, and factors correlated to food insecurity in Australian university students is important. The current study aimed to: (i) assess the correlates of food insecurity amongst university students in Western Australia; (ii) explore the levels of severity of food insecurity by psychological distress and enrolment types; (iii) identify the difference in the severity of food insecurity between students with and without children; and (iv) assess food assistance usage amongst university students in Western Australia.

## 2. Materials and Methods

### 2.1. Study Participants

Undergraduate and postgraduate students enrolled at one Western Australia metropolitan university campus were eligible to complete a survey. A total of 52,639 students were enrolled in 2020, the largest university enrolments in Western Australia [39,40]. Fifty-seven percent of the students were female, and 26% were international students at the time of the research [39]. Those who were under 18 years old and not accessing their OASIS account during the data collection period were not included in this study.

### 2.2. Study Design

A cross-sectional survey was conducted between 20 July and 25 September 2020. A non-random sample of students was recruited using a weblink promoted through social media, on the student services website homepage, and at the Student Guild site that offers food assistance. Participants completed an online, self-administered Qualtrics XM (SAP., Seattle, WA, USA) survey. The link directed respondents to a participant information form, and consent was obtained prior to commencing the survey.

**Figure 1:**
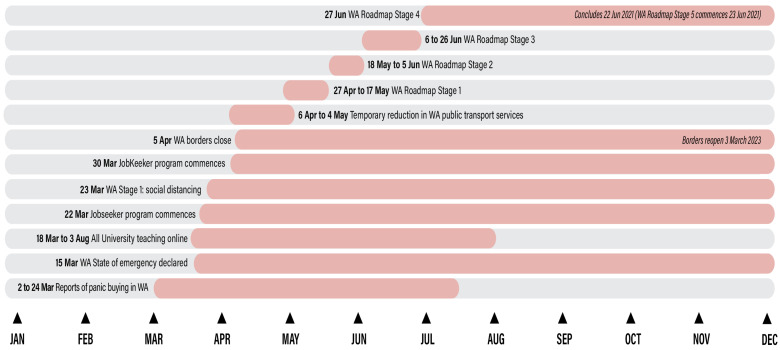
Timeline of Western Australian COVID-19 responses and university responses.

A total sample of n = 213 current students completed the survey. This study followed the STROBE guidelines (a checklist of items to address in articles reporting an observational study) [41] and was approved by Curtin University Human Research and Ethics Committee (Approval number HREC2020-0373). A timeline of major COVID-19 mitigation policies enacted in Western Australia during 2020, where the current study was located, is depicted in Figure 1 [42]. The policy enactments required ‘stay at home’ measures for people not involved in essential services and reduced employment, particularly for casual workers. In line with State Government requirements, Curtin University teaching models moved fully online on 18 March 2020 so that the students who could return were sent to their homes for a time. Not all international students were able to return to their home county in the early stages of the pandemic due to international border closures and preferred to stay in WA [43]. Teaching was partially resumed on campus in August 2020, with the two-square-meter rule maintained in all learning spaces. Students outside of Western Australia were still unable to travel to the state because of ongoing severe border restrictions and hence continued to receive support to study online [43].

### 2.3. Measures

The survey instrument comprised questions in four areas: socio-demographic, experience of food insecurity, psychological distress, and access to food assistance. The socio-demographic characteristics include age, gender, country of birth, ethnicity, main language spoken at home, enrolment type, degree type, faculty of study, and whether they have children. Students also self-reported their weight and height to calculate BMI (kg/m^2^).

Food insecurity was measured using the 18 item United States Department of Agriculture Household Food Security Survey Module (USDA HFSSM), a reliable and validated tool that measures the extent and experiences of food insecurity and hunger [31,41]. HFSSM’s 18 questions capture and distinguish the various levels of severity of food insecurity and take into account the impact on dependent children [26]. The time period used in the instrument was modified to incorporate changes since COVID-19 began (e.g., since 20 March 2020), and responses were analyzed in accordance with the USDA Guide to Measuring Household Food Security [26,27,44]. In brief, food security status was categorized based on raw scores of the items asked, and if there are children in the household, eight child-related questions were included in the raw score. For students with one or more children, out of the maximum raw score of 18, a raw score of zero was classified as high food security, 1–2 as marginal food security, 3–7 as low food security, and 8–18 as very low food security [26,44]. For students without children, out of the maximum raw score of 10, a raw score of zero was classified as high food security, 1–2 as marginal food security, 3–5 as low food security, and 6–10 as very low food security [26,44]. Students with high or marginal food security were grouped as ‘food secure’ while those with low or very low food security were grouped as ‘food insecure’ [44].

Levels of psychological distress across three axes (depression, anxiety, and stress) were assessed using the reliable and validated 21 item Depression, Anxiety, and Stress Scale (DASS-21) [45,46]. The purpose of the scale is to assess the severity of core groups of depression, anxiety, and stress and is not to diagnose psychological-related distress [45]. The items measured the prevalence of symptoms of depression, anxiety, or stress with a four point Likert scale (ranging between 0 = Never and 3 = Almost always). In brief, each area was classified into five levels, indicating normal (0), mild (1), moderate (2), severe (3), and extremely severe (4) problems. The scores for depression, anxiety, and stress each ranged from 0 to 42 (multiplying summed scores by 2) [45]: For depression, a total score of 0–9 was classified as normal, 10–13 as mild, 14–20 as moderate, 21–27 as severe, and 28–42 as extremely severe. For anxiety, a raw score of 0–7 was classified as normal, 8–9 as mild, 10–14 as moderate, 15–19 as severe, and 20–42 as extremely severe. For stress, a score of 0–14 was classified as normal, 15–18 as mild, 19–25 as moderate, 26–33 as severe, and 35–42 as extremely severe [45].

Students were also asked if they had accessed food assistance during the official lockdown period. Those accessing food relief were asked where they sourced the food from [response options: friends and/or family, student guild food parcels, church community, voucher charity, emergency relief, and/or food hampers charities].

### 2.4. Statistical Analyses

Descriptive analyses were performed, and the frequencies and proportions for categorical variables and the means and standard deviation for continuous variables were reported. Univariate logistic regression models were conducted to test the association between food insecurity and relevant variables to generate an unadjusted odds ratio. All variables that were found to have a *p* ˂ 0.1 in the univariate regression were entered into the multivariable logistic regression model to generate adjusted odds ratios (AOR) for food insecurity. The significance level for multivariable logistic regression was set at *p* ˂ 0.05. The levels of severity of food insecurity among students with and without children were compared using the Chi-square test for categorical variables (e.g., enrolment type), and ANOVA tests were used for continuous variables (e.g., depression score). Analyses to assess the association between food insecurity and examined factors were conducted on 211 participants, excluding two incomplete datasets. Stata SE version 17 was used to conduct all data analyses.

## 3. Results

### 3.1. Socio-Demographics and Enrolment Status

Table 1 outlines the total sample socio-demographic characteristics (n = 213). Seventy percent (n = 149) of respondents were female, and the mean age was 26.07 years (SD = 7.02; age range = 18–53 years). Fifty-eight percent (n = 125) of respondents were born outside of Australia, with the highest proportion identifying as having Asian background (52%; n = 111). Forty-nine percent (n = 111) were enrolled as international students, and 56% (n = 119) were completing an undergraduate degree, 34% (n = 72) were in the Faculty of Health Sciences.

### 3.2. Factors Associated with Food Insecurity

Overall, 48% (n = 101) of respondents reported experiencing food insecurity. The univariate logistic regression analyses indicated that gender, country of birth, the main language spoken at home, ethnicity, enrolment type, degree type, degree year, faculty, and mental health status (depression, anxiety, and stress) were associated with experiencing food insecurity (*p* < 0.1).

All variables that were statistically significantly associated with food insecurity at *p* < 0.1 identified in the univariate analyses were entered into the multivariable logistics regression model and experienced a decrease in strength, except for the enrolment type and depression levels. The adjusted model indicates that the likelihood of experiencing food insecurity among international students was ninefold that of domestic students enrolled full-time (AOR 9.13; 95% CI: 2.32–35.97; *p* = 0.002) after controlling for all the variables that emerged significant in univariate analyses.

The multivariable model also indicated that depression was significantly positively associated with food insecurity, with those reporting a one unit higher level of depression being 1.62 times more likely to experience food insecurity (AOR 1.62; 95% CI: 1.12–2.33; *p* = 0.010). For example, students reporting moderate depression levels were 1.62 times more likely to experience food insecurity compared to those reporting mild depression levels. Potential interactions between relevant variables were tested and found to be non-significant; hence, no interaction term was included in the model. See Table 2 for the results of univariate and multivariable logistics regression analyses assessing factors associated with food insecurity among university students.

### 3.3. The Severity of Food Insecurity among Students with Children

Table 3 expands on the severity of food insecurity experienced by psychological distress and student enrolment type through further analysis among students with and without children. Of the overall respondents who reported experiencing food insecurity (n = 101), 48% (n = 49) experienced low food insecurity, and 51% (n = 52) experienced very low food insecurity. A significant difference in the levels of severity of food insecurity was found between international versus domestic students, regardless of having children (*p* ˂ 0.001 for both with and without children). From a total of 52 respondents who experienced very low levels of food security (severe food insecurity), 83% (n = 43) were international students.

Twenty-six percent of respondents (n = 56) reported that they were parents of dependent children; of these, 35% (n = 21) were international student parents. Of the international students with children, 90% (n = 19) experienced food insecurity (low and very low food security) compared to 20% of domestic students with children (n = 7) (*p* < 0.001). Overall, higher levels of very low food security were reported among students with children, particularly the highest prevalence of severe food insecurity was reported among international students with children (57%) compared to their counterparts without children (38%) and domestic students with (6%) and without children (9%), *p* < 0.05 for all comparisons.

### 3.4. Severity of Food Insecurity and Psychological Distress

Self-reported psychological distress differed significantly by the level of severity of food insecurity. The average depression score was higher among students who experienced very low food security compared to all three less severe categories of food security (χ^2^ = 12.11, *p* < 0.001). Compared to students with high food security, students who experienced very low food security reported higher mean scores of depression (mean = 21.45, SD = 11.631 vs. mean = 9.77, SD = 9.34; *p* < 0.001), anxiety (mean = 15.57, SD = 10.14 vs. mean = 7.10, SD = 8.60; *p* < 0.001), and stress (mean = 18.78, SD = 10.89 vs. mean = 13.15, SD = 10.09; *p* = 0.029). Likewise, compared to students experiencing marginal food security, those experiencing very low food security reported higher depression (mean = 21.45, SD = 11.31 vs. mean = 14.46, SD = 11.45; *p* < 0.001) and anxiety levels (mean = 14.63, SD = 9.81 vs. mean = 10.23, SD = 9.49; *p* < 0.001). Table 3 shows the levels of food insecurity by psychological distress.

### 3.5. Food Assistance

Forty-seven percent of respondents (n = 100) reported needing support to access food since the beginning of the COVID-19 pandemic. Of these, 59% (n = 59) obtained food support from their friends and/or family, 29% (n = 29) accessed Student Guild food parcels, and 15% (n = 15) received food hampers from charity organizations (e.g., Salvation Army, Anglicare, Second Bite, St Vincent Paul and Foodbank). Thirty-one percent of students who indicated that they needed food relief (n = 31, 31%) obtained support from more than one food relief source. Of students who needed food assistance (n = 100), 29% (n = 29) were food-secure students, and about three-quarters of them (n = 21) accessed food from their friends and/or family. Table 4 shows the support students used to access food by the level of severity of the experience of food insecurity.

## 4. Discussion

The current study assessed the factors associated with food insecurity among university students in Western Australia during the early stages of the COVID-19 pandemic in 2020. This study examined food insecurity correlates and the difference in the severity of food insecurity between students with and without children. About half of the university students surveyed in the current study experienced food insecurity, a higher rate than the general population. The high prevalence of food insecurity among university students has also been reported in previous pre-pandemic studies ranging from 13–48 percent [9,21,22], and the current study, although not a prevalence study, found a rate of food insecurity consistent with the higher-end. This may be partly driven by changes in living arrangements, loss of employment, and/or travel restrictions as a result of the COVID-19 pandemic [47,48].

International students, those from other countries who were studying in Western Australia, were nine times more likely to experience low or very low food security than domestic students in the current study, similar to the higher prevalence of food insecurity among temporary residents in Australia previously reported [7,24,25]. For example, a Tasmanian study reported that temporary residents had fourfold increased odds of experiencing food insecurity compared with those who were Australian citizens [7].

The current study’s findings suggest that Australian university students, particularly non-residential students, may have been disproportionately impacted during the COVID-19 pandemic. Numerous factors may account for higher rates of food insecurity among international students in the Australian context. These include high levels of hospitality and accommodation industry job losses resulting from COVID-19 lockdown measures (e.g., stay-at-home orders and business closures) impacting international students’ ability to work in these industries [7,49]. While Australian governments provided some assistance to residents in the form of welfare assistance payments (e.g., Job Keeper and Job Seeker), guaranteeing funds for domestic students, it did not support temporary residents [50]. This is concerning given Australia has the highest ratio of international students per head of population in the world, comprising 21% of all Australian university enrolments in 2019 compared to 6% or less in other countries [51].

There was a higher prevalence and severity of food insecurity among university students with children in this study. The severity of food insecurity was greater among international university students with children than those without children or domestic students with and without children. While Australian studies reported a higher prevalence of food insecurity among international university students than domestic students [24,25], they did not assess food insecurity among parents. The severity of food insecurity among students with dependent children is a concern, given student parents comprise a significant portion of university populations, 27% of the current study participants, and 13% nationally in the 2016 Australian census survey [52]. This finding highlights the need for particular attention to be given to student parents due to the negative impacts of food insecurity on the students themselves as well as on their children [52,53,54]. Parents experiencing food insecurity may compromise their own nutritional intake to preserve the adequacy of their children’s diets [55,56,57], which can impact their own academic achievement, physical health, mental well-being, and social operations [57,58].

Depression was significantly positively associated with the experience of food insecurity in the current study, with higher levels of depression and anxiety reported among students who experienced very low food security than those who were food secure. Although a causal relationship between mental health problems and food insecurity cannot be drawn from the current study, there is existing evidence that the experience of not being able to access food or hunger is traumatizing or associated with worry, depression, and anxiety. North American studies reported that food-insecure graduate students face higher levels of depression and anxiety, underlining that food insecurity is positively associated with mental well-being among university students [22,32,33]. A previous Western Australian government surveillance study shows an increased likelihood of high levels of psychological distress associated with food insecurity [6]. Collectively, the findings of this study, along with previous studies among Australian university students, highlight food insecurity as an emerging problem among university students and associated with mental health problems, suggesting future food security initiatives are trauma-informed and incorporate strategies to support mental well-being, particularly among international students and those with children.

About half (48%) of the participants in the current study had sought some form of food assistance, yet only 29% were categorized as food secure, suggesting that the food assistance was insufficient to mitigate the experience of food insecurity among this cohort. The use of multiple sources of food relief (e.g., from family, friends, charities, and student guild) also supports this. Food relief initiatives in Australia usually provide short-term food relief, and previous research has identified the inadequacies in the system [59]. On the other hand, almost a third of students who experienced food insecurity did not access food assistance. This may be partly driven by the social stigma associated with accessing food assistance, which has been described as undignified and socially unacceptable in a country as wealthy as Australia [60]. The unique context of the early stages of the COVID-19 pandemic saw food relief services having to pivot their activities due to the loss of volunteers, disruptions in food supplies and distribution networks, and physical distancing directives. Communications regarding food assistance may not have reached some students who suddenly found themselves physically and socially isolated and unable to access food retail. Learning from the experience of COVID-19, administrators should seek to improve communication channels for students, particularly international students. There was a lack of awareness among food relief organizations of sub-population groups who were particularly vulnerable to food insecurity in the early stages of the pandemic. The university Student Guild was approached by increasing numbers of students in distress and needing food. They then had to find additional sources of food and approach one of the academics who was known to work in the area of food insecurity (CMP), who was then able to connect them with emergency food relief organizations that were distributing food hampers that had been originally donated by a major supermarket to respond to food shortages due to the catastrophic Australian bushfires in December 2019.

The strengths of the current study include the use of reliable and validated 18 item USDA HFSSM measure to enable a comprehensive examination of the severity and impact of food insecurity and to examine the potential impact on dependent children, not possible with the instruments used in previous studies [6,22]. In addition, this is the only study the authors are aware of that measures the extent and severity of food insecurity among Australian university students comparing those with and without children. Another strength is the use of the DASS-21, a reliable and validated psychological distress assessment tool, to measure levels of depression, anxiety, and stress. Furthermore, this study provides an overview of food insecurity among a sample of Western Australian university students. This is an opportunistic study conducted during the early stages of the COVID-19 pandemic, and food insecurity has not been measured in a Western Australian university population previously.

There are several potential limitations that should be considered when interpreting the findings of the current study. Given the recruitment method and low response rate, the findings may not be generalizable to the wider university student body or other university populations and should be interpreted with caution. Given the survey link was widely distributed among the University students, appearing on the University Student Oasis homepage and at the Student Guild site that offers food assistance, participation bias is likely to have occurred. This is due to the likelihood that participating in the survey meant a higher relation with food insecurity. Although Australian universities have experienced a decline in the number of international students since the start of the pandemic [61], there was a greater proportion of international student participants in the current study than those who were enrolled at the studied university in 2020 (49 vs. 26%) [61]. This may be due to the non-residential students who were in Australia being disproportionately impacted during the COVID-19 pandemic, and they may have been more likely to be interested in participating [47,48]. Research exploring the prevalence of food insecurity among university students using a representative sample is recommended to provide a robust estimate of the problem going forwards. This study relied on self-reported data, which may be subject to social desirability bias and recall bias; however, the self-reported 18 item FSSM instrument was chosen to measure food security which is considered the gold standard [26,27]. In addition, the cross-sectional study design limits the ability to draw conclusions about causal relationships between the examined factors and food insecurity. Routine monitoring of the population prevalence of food insecurity is recommended with an appropriate sample size to enable exploration of the causes and consequences of food insecurity among Australian university students, including mental and physical health problems. Income, a crucial determinant of food insecurity, was not measured in the current study and is a limitation of this research and should be incorporated into future research. As a proportion of students accessing food relief were food insecure, a diet quality measure is recommended as well. This information will be useful in informing fit-for-purpose food assistance, as understanding students’ dietary patterns will enable tailoring of food procurement to suit their dietary practice, particularly for international students.

Despite the limitations specified in the current study, several implications can be drawn for future practice and advocacy for a policy change in Australian universities. These current findings, along with recent research, support a call for an *Australian University Food Security Framework* that requires universities to have a food security strategy and implementation plan [62], similar to the Australian University Mental Well-being Strategy [63,64]. Fit-for-purpose policy and interventions, including problem definition (identifying and responding to individual students and/or groups of students who are at increased risk of food insecurity), are warranted, particularly for the welfare of international students and students who are parents. Inviting students to be involved in co-designing effective interventions would be beneficial to improve food insecurity and mental health problems among university students [65].

Future public health emergency disaster preparedness and response planning may continue to impact food access, particularly as the frequency of disasters increases. The declaration of the COVID-19 pandemic as a public health emergency and the mitigation strategies had various unintended impacts, including food supply chain disruptions, loss of employment and income, and restricted public movement, all of which impacted household food security. Insights gained from research undertaken during the early stages of the COVID-19 pandemic and other disasters can be used to help inform future interventions and policies. The continuing impact related to unstable finances and increasing cost of living expenses (e.g., food, housing, and education costs) needs to be considered in preparing strategies to assist university students, particularly non-residential students. These findings add to the growing body of evidence regarding the social impacts of the COVID-19 pandemic on vulnerable population sub-groups, highlighting the need for social policy reforms. Currently, Australian universities support students with emergency food relief or supermarket vouchers; however, the food provided is unlikely to meet the social and nutrition needs of this vulnerable sup-population group [66]. The COVID-19 pandemic response did not adequately support vulnerable students, suggesting that there is a gap in university planning for disaster responses. Holistic, effective, and integrated disaster management plans to reduce the risk of food insecurity targeted at both residential and non-residential students are needed. As with all effective disaster response planning, the first stage would be to identify and document the existing capacity to cope with food shortages and create a mitigation plan. Given the results of the current study, the plan should consider financial support and emergency food relief tailored to students’ cultural needs. University-managed and funded free or subsidized meals would be a dignified response to food shortages during the early stages of an emergency. Scenario planning based on the estimated number of students likely to be impacted during an emergency would support effective disaster response in the future [24,67].

Finally, routine surveillance of the experience of food insecurity and psychological distress among Australian university students is crucial for monitoring and reporting university students’ welfare [62]. This would support university bodies in navigating potential initiatives and developing feasible strategic plans to address food insecurity and psychological distress among students. It is particularly important to monitor the human right to food among this vulnerable population group and to encourage educational participation and attainment. Both the human right to food and the quality of education are important strategies for achieving Sustainable Development Goals that contribute to economic development and well-being [68].

## 5. Conclusions

The current study’s findings show that since the COVID-19 pandemic in 2020, the prevalence and severity of food insecurity have been high among university students surveyed, particularly among international students and those with children. Higher levels of depression and anxiety were also reported among university students experiencing food insecurity. This research findings describe the experience of food insecurity in a sample of university students during the early stages of the COVID-19 pandemic. Given the ongoing impact of the COVID-19 pandemic and the increasing likelihood of disaster events impacting the food supply in Australia, and the psychological distress associated with food insecurity, further research is recommended to routinely monitor food insecurity in a representative sample of university students.

## Figures and Tables

**Table 1:** Socio-demographic characteristics of university student respondents (n = 213).

Demographic Variable	n	%
Age range		
18–24	112	53
25–34	73	34
35–44	21	10
45–54	7	3
Mean (SD)	26.07 (7.02)	
Age range	18–53 years	
Gender—Female	149	70
Country of Birth		
Australia	88	41
Other	125	59
Main language at home		
English	122	57
Other	91	43
Ethnicity		
Caucasian	77	36
Asian	111	52
Black or African American	4	2
Mixed/multiple ethnicities/Other	19	9
Prefer not to say	2	1
Current student enrolment type		
International	104	49
Full-time domestic	93	44
Part-time domestic	16	7
International students’ Visa Status (n = 104)		
Student visa—Temporary Migrant	97	93
Other	7	7
Degree type		
Undergraduate	119	56
Postgraduate course work	69	32
Higher degree by research (Master, PhD)	25	12
Year of Study		
First year	70	33
Second year	80	38
Third year or later	63	29
Faculty of study		
Health Sciences	72	34
Business and Law	58	27
Science and Engineering	45	21
Humanities	38	18
Smoking status—Smokers	16	8
Student with child(ren)	56	26
Weight status		
Underweight	11	5
Normal weight	138	65
Overweight	64	30
Depression score—Mean (SD)	14.81 (11.49)	
Anxiety score—Mean (SD)	10.53 (9.70)	
Stress score—Mean (SD)	15.27 (10.70)	

Note: SD = Standard Deviation.

**Table 2:** Univariate and multivariable logistic regression assessing factors associated with food insecurity, Western Australia 2020 (n = 211).

Variables	Univariate		Multivariable	
	OR (95% CI)	*p*-Value ^^^	AOR (95% CI)	*p*-Value
Age	1.02 (0.98–1.06)	0.418	-	-
Male	Ref	Ref	Ref	Ref
Female	0.49 (0.27–0.89)	0.019	0.63 (0.27–1.46)	0.279
English language at home	Ref	Ref	Ref	Ref
Other	5.36 (2.95–9.72)	<0.001	0.92 (0.33–2.59)	0.878
Australia country of birth	Ref	Ref	Ref	Ref
Other	7.26 (3.86–13.65)	<0.001	2.21 (0.52–9.40)	0.282
Full-time domestic enrolment	Ref	Ref	Ref	Ref
Part-time domestic enrolment	0.79 (0.21–3.04)	0.733	0.80 (0.17–3.68)	0.772
International enrolment	13.35 (3.52–50.67)	<0.001	9.13 (2.32–35.97)	0.002
Undergraduate degree	Ref	Ref	Ref	Ref
Postgraduate degree	2.66 (1.52–4.66)	0.001	1.04 (0.43, 2.53)	0.934
First year of degree	Ref	Ref	Ref	Ref
Second year of degree	1.96 (1.02–3.76)	0.044	1.32 (0.54–3.24)	0.544
Third year or later of degree	0.84 (0.42–1.69)	0.629	0.63 (0.25–1.59)	0.327
Health Sciences faculty	Ref	Ref	Ref	Ref
Business and Law faculty	1.89 (0.92–3.87)	0.081	1.15 (0.44–3.00)	0.771
Science and Engineering faculty	3.55 (1.62–7.77)	0.002	1.78 (0.60–5.28)	0.302
Humanities faculty	2.18 (0.97–4.86)	0.058	2.52 (0.89–7.12)	0.082
Caucasian	Ref	Ref	Ref	Ref
Asian	6.87 (3.52–13.40)	<0.001	0.56 (0.12–2.65)	0.469
Mixed/Others	3.26 (1.26–8.44)	0.015	0.77 (0.19–3.07)	0.707
Non-smoker	Ref	Ref	-	-
Smoker	0.69 (0.25–1.94)	0.487	-	-
Normal BMI	Ref	Ref	-	-
Underweight BMI	1.35 (0.39,4.64)	0.634	-	-
Overweight and obesity BMI	1.06 (0.58,1.92)	0.855	-	-
Depression level	1.58 (1.29–1.94)	<0.001	1.62 (1.12–2.33)	0.010
Anxiety level	1.42 (1.18–1.71)	0.004	1.11 (0.79–1.55)	0.555
Stress level	1.19 (0.96–1.46)	0.112	-	-

Note: Ref: Reference Category; OR: Odds Ratio; SE: Standard Error; 95% CI: Confidence Interval; AOR: Adjusted Odds Ratio; ^^^ *p* > 0.1 in univariate analyses are excluded from the multivariable logistics regression model; ‘-’ indicates a non-significant association for univariate logistic regression analyses.

**Table 3:** Severity of food security among university students by enrolment type and psychological distress (n = 211).

	Total	Food Security Category				χ^2^-Test	*p*-Value
		High	Marginal	Low	Very Low		
	n (%)	n (%)	n (%)	n (%)	n (%)		
Students without children (n = 155)						49.91	˂0.001
Domestic	74 (48)	44 (59) ^1^	13 (18) ^1^	10 (14) ^1^	7 (9) ^1^		
International	81 (52)	7 (9) ^2^	16 (20) ^1^	27 (33) ^2^	31 (38) ^2^		
Students with children (n = 56)						28.39 *^^^*	˂0.001
Domestic	35 (62)	20 (57) ^1^	8 (23) ^1^	5 (14) ^1^	2 (6) ^1^		
International	21 (38)	1 (5) ^2^	1 (5) ^1^	7 (33) ^1^	12 (57) ^2^		
	**M (SD)**	**M (SD)**	**M (SD)**	**M (SD)**	**M (SD)**	***F-*** **Test**	***p-*** **Value**
Depression	14.81 (11.49)	9.77 (9.34) ^a^	14.46 (11.45) ^ab^	15.31 (10.84) ^b^	21.45 (11.31) ^c^	12.11	<0.001
Anxiety	10.53 (9.70)	7.10 (8.60) ^a^	10.23 (9.49) ^a^	10.29 (8.51) ^a^	15.57 (10.14) ^b^	8.53	<0.001
Stress	15.27 (10.70)	13.15 (10.09) ^a^	15.60 (10.89) ^ab^	14.29 (10.08) ^ab^	18.78 (10.89) ^b^	3.06	0.029

Note: *p*-value set at ≤0.05. ^ Fisher’s Exact test as some of the cell counts are <5. Different superscript numbers denote significant differences in the proportions of the severity of food insecurity between enrolment types. Different superscript letters denote significant differences in the mean scores between levels of severity of food insecurity.

**Table 4:** Support used to access food by the severity of food insecurity (n = 211).

Variable	Total	Food Security Category				χ^2^/Fisher’s Exact Test	*p*-Value
		High (n = 72)	Marginal (n = 38)	Low (n = 49)	Very Low (n = 52)		
	n (%)	n (%)	n (%)	n (%)	n (%)		
Supported to access food (n = 211)							
Yes (all sources)	100 (47)	8 (11) ^a^	21 (55) ^b^	31 (63) ^b^	40 (77) ^c^	40.76	<0.001
No	111 (53)	64 (89)	17 (45)	18 (37)	12 (23)		
Accessed food relief (Yes) ^^^	**(n = 100)**	**(n = 8)**	**(n = 21)**	**(n = 31)**	**(n = 40)**		
Friends and/or family	59 (59)	7 (12)	14 (24)	17 (29)	21 (36)	4.12	0.249
Guild food parcels	29 (14)	1 (13)	4 (19)	9 (29)	15 (38)	3.47	0.324
Church Community	11 (5)	0 (0)	3 (14)	6 (19)	6 (15)	1.88	0.597
Voucher Charity	15 (7)	0 (0)	1 (5)	3 (10)	7 (18)	3.61	0.307
Emergency relief	13 (6)	0 (0) ^a^	1 (5) ^a,b^	2 (6) ^b^	10 (25) ^c^	8.72	0.033
Charity food hampers	9 (4)	0 (0)	1 (5)	2 (6)	6 (15)	3.26	0.354

Note: The Fisher exact test was conducted if the expected count is less than 5. Different superscript letters in a row denote significant differences in the proportion of students supported to access food across the level of severity of food insecurity. ^ Frequencies can up to more than 100 as students accessed multiple food assistance support sources.

## Data Availability

Data is unavailable due to privacy reasons.
